# A general sample size framework for developing or updating a predictive algorithm: with application to clinical prediction models

**DOI:** 10.1186/s12874-026-02856-7

**Published:** 2026-04-29

**Authors:** Richard D. Riley, Rebecca Whittle, Mohsen Sadatsafavi, Glen P. Martin, Alexander Pate, Gary S. Collins, Joie Ensor

**Affiliations:** 1 Department of Applied Health Sciences, School of Health Sciences, College of Medicine and Health, University of Birmingham, Birmingham, UK; 2 National Institute for Health and Care Research (NIHR) Birmingham Biomedical Research Centre, Birmingham, UK; 3 Respiratory Evaluation Sciences Program, Faculty of Pharmaceutical Sciences, The University of British Columbia, Vancouver, Canada; 4 Division of Informatics, Imaging and Data Science, Faculty of Biology, Medicine and Health, University of Manchester, Manchester Academic Health Science Centre, Manchester, UK

**Keywords:** Predictive algorithms, Clinical prediction models, Sample size, Penalised regression, Lasso and ridge regression

## Abstract

**Background:**

When developing or updating a predictive algorithm to make predictions (e.g., risk estimates) in individuals, the sample size of the development (training) dataset is an important consideration. Small datasets reduce a model’s predictive performance, generalisability and fairness, and may lead to harm. To help identify (minimum) sample sizes required for model development or updating, various sample size calculations exist but their underlying theory is based on standard (unpenalised) regression and focus on minimising overfitting. To address this, we propose a more general approach, which allows extension to any machine learning or AI-based modelling method, and to any performance metric (estimand) of interest.

**Methods:**

The proposed approach draws samples from anticipated posterior distributions to examine the impact on degradation in a model’s predictive performance compared to a reference model. It requires researchers to provide candidate predictors (features), a reference model (e.g., based on mean outcome incidence, predictor weights and c-statistics of previous models), and a (existing, synthetic or pilot) dataset reflecting the joint distribution of candidate predictors in the target population. A fully simulation-based approach then generates thousands of models conditional on a chosen sample size and model development strategy, to produce posterior distributions of individual predictions and model performance (degradation) metrics, to inform required sample sizes. To improve computational speed for penalised regression, we also propose a one-sample Bayesian approximation that combines shrinkage priors with a likelihood decomposed into sample size and Fisher’s unit information.

**Results:**

The approaches are illustrated when developing models for pre-eclampsia using logistic regression (unpenalised, uniform shrinkage, lasso or ridge) and random forests; and are shown to encompass existing sample size calculation criteria whilst also providing model assurance probabilities, instability metrics, and degradation statistics about calibration, discrimination, clinical utility, prediction error and fairness. Uncertainty in the reference model can also be accounted for in the calculations.

**Conclusions:**

Our approach provides a general framework for (minimum) sample size calculations for developing or updating a prediction model, and is applicable for any statistical, machine learning or AI-based development method for supervised learning. Crucially, the recommended (minimum) sample size will depend on users’ chosen estimands and model development (or updating) approach. Example R and Stata code is provided.

**Supplementary Information:**

The online version contains supplementary material available at 10.1186/s12874-026-02856-7.

## Introduction

Studies that develop or train a predictive algorithm use a sample of data, representative of a target population (e.g., women with breast cancer), to produce a model for forecasting an individual’s outcome (e.g., predicted 5-year risk of cancer recurrence) conditional on their values of multiple features or predictors (e.g., age, stage of disease). An example is the ISARIC model [1], for use in hospitalised adults with COVID-19 to estimate their risk of in-hospital clinical deterioration based on 11 predictors measured at hospital admission. In the healthcare setting, which is the applied focus of this article, predictive algorithms are better known as clinical prediction models (CPMs).

Various papers have proposed minimum sample size calculations for studies developing (training) a CPM, to help improve their robustness, generalisability, predictive performance and clinical utility [2]. Initially these stemmed from rules-of-thumb [3], but recently more tailored equations and approaches have emerged for determining the sample size required to target precise predictions, low overfitting and instability, small mean square prediction error, and a small loss of clinical utility [4–11]. However, this body of work is mainly based on standard (unpenalised) regression-based approaches, and so in this article we propose a more general framework for sample size calculations to develop or update a CPM using any predictive AI-based modelling approach (statistical or machine learning) for supervised learning. Specifically, we bring together various well-established statistical concepts, approaches and metrics, to provide researchers with an integrated framework to identify the minimum sample size required to develop a model that targets some pre-specified performance criteria.

Briefly, our proposal anticipates the sampling (posterior) distributions of individual-level predictions for a particular sample size, in the context of a particular model development strategy and included set of candidate predictors. Then, by repeated sampling from these distributions, the impact of a particular sample size can be examined in terms of model performance metrics. These may include measures of: (i) *model degradation*, which summarise the anticipated reduction in model performance (e.g., calibration, discrimination and clinical utility of estimated risks) compared to an assumed reference model (e.g., based on previous models in the field); (ii) *prediction instability*, for example as defined by the mean width of 95% uncertainty intervals around individual risk estimates; and (iii) *model assurance*, defined by the probability that a single dataset will give a CPM that meets some pre-defined criteria (e.g., calibration slopes between 0.9 and 1.1; relative value of sample information for net benefit > 90%).

The article outline is as follows. The Methods section describes the overall premise and requirements for minimum sample size calculations for CPM development and then outlines our general simulation-based approach to examine model degradation, instability and assurance relative to a reference model. To improve computational time, an approximation is proposed for Bayesian penalised regression based on combining shrinkage priors with a decomposition of Fisher’s information matrix. The Results section applies the approaches to examine the minimum sample size required for developing CPMs for adverse outcomes in pre-eclampsia. The Discussion section concludes with some discussion. Although we focus on CPMs, the proposed methods are applicable to any setting where predictive algorithms are needed in individuals.

## Methods

We begin by describing the premise of sample size calculations for CPM development, as this forms the bedrock of our new approaches subsequently outlined. Our focus is CPMs for binary outcomes (0 = no event, 1 = event). Throughout, we refer to model *development* but the framework naturally covers model *updating* (a special case of model development).

### Premise of sample size calculations for CPM development studies

Sample size considerations for CPM development studies are very different than for traditional medical research studies (e.g., trials); whereas those focus on statistical power and minimising type I and II errors, CPM development studies should rather focus on sample size to improve the robustness and performance of model predictions, as now explained.

- The calculation should address the key estimand of the study

When developing a CPM for a binary outcome conditional on a set of candidate predictors, the estimand of interest is the risk (probability) of the outcome event for each individual in the target population. Therefore, for the sample size calculation, the focus is on how sample size impacts a CPM’s sampling or posterior distribution of the outcome risk (probability) for each individual in the target population. A sampling/posterior distribution for individual risk reflects the information arising from the CPM, for example in terms of the mean (best estimate of risk) and uncertainty (e.g., 95% range of plausible risks).

In this article, we refer to uncertainty distributions (i.e., sampling and posterior distributions) broadly as posterior distributions, and define the $i$^th^ individual’s posterior distribution by $\mathrm{p}\left({p}_{i} \right|\boldsymbol{ }{\boldsymbol{\upbeta}},{{\boldsymbol{x}}}_{{\boldsymbol{i}}})$ where ${\boldsymbol{\upbeta}}$ refers to the parameters of some true (but unknown) CPM, ${{\boldsymbol{x}}}_{{\boldsymbol{i}}}$ denotes the individual’s values of the predictors in the model and ${p}_{i}$ denotes the individual’s true (underlying) risk conditional on ${\boldsymbol{\upbeta}}$ and ${{\boldsymbol{x}}}_{{\boldsymbol{i}}}$.

(b) The calculation should have target values for statistical performance and assurance

Small sample size reduces model performance in terms of overall fit, calibration, discrimination and clinical utility, and leads to large uncertainty in individual predictions (reflecting model instability) [12]. In other words, smaller sample sizes increase epistemic uncertainty (model-based error) and degrade (i.e., worsen) a model’s performance in the target population compared to if we knew the model perfectly (no model-based error). In this paper, we refer to this concept as *model degradation*. In the target population of interest, and for the specific set of predictors considered in the model development, conceptually we define model degradation as,

$$\begin{aligned}\mathbf{model\,degradation}=&\boldsymbol(\mathbf{performance}\;\mathbf{of}\,\mathbf{developed}\,\mathbf{model}\boldsymbol)\\&-(\mathbf{performance}\boldsymbol\;\mathbf{of}\boldsymbol\;\mathbf{true}\boldsymbol\;\mathbf{model}\boldsymbol)\end{aligned}$$

where the performance of the true model (as defined by a chosen metric, see below) is that which would be obtained if we had perfect information (i.e., an infinite sample size) about the predictor-outcome relationships [6]. Related concepts are the loss function and value of perfect information [13]. Note that degradation is a different (albeit related) concept to model optimism. Optimism refers to the difference (usually caused by model overfitting) between model performance in the development data and the target population [14]. In contrast, degradation is about the difference in performance between a fitted model and a true model, when both are applied in the target population. Of course, fitted models that have more optimism are likely (other things being equal) to have more degradation, so these issues are related.

The sample size for a model development study should target an acceptable level (as defined by the researchers in their context of interest) of *model degradation*, in terms of the performance measures defined in Tables 1 and 2. A model degradation value of zero indicates no degradation. However, the formal interpretation of non-zero values is dependent on the specific performance metric being used in the calculation. For example, values of the c-statistic (a measure of discrimination) and net benefit (a measure of clinical utility [16]) decrease when model performance decreases; thus, their model degradation values must be $\le$ 0. Conversely, for the mean absolute predictor error (MAPE), model degradation values must be $\ge$ 0 as MAPE for a developed model must be $\ge$ the true model’s MAPE value of 0. Further, for the calibration slope (true model value = 1), model degradation can have positive or negative values, as a developed model may exhibit systematic over-prediction (calibration slope < 1) or under-prediction (calibration slope > 1).

**Table 1:** Predictive performance measures of interest for examining expected model degradation and assurance probabilities when using a particular sample size for CPM development

Compared to the predictions and performance of a ‘true model’ (i.e., the model that would be obtained had we an infinite sample size to apply our chosen development approach on the same set of candidate predictors), a CPM development study may desire a sample size that targets acceptable levels for the following measures: • **reduction in the percentage of explained outcome variation in the target population**; for example, as measured by R^2^ (percentage of outcome variance explained by the model), including generalised R^2^ measures for binary outcomes such as the Cox-Snell or Nagelkerke R^2^ • **reduction in discrimination performance in the target population;** discrimination measures the separation of a model’s estimated risks between those with and without the event, typically measured by the c-statistic (equivalent to AUROC for binary outcomes) • **mean square or absolute difference in estimated and ‘true’ model predictions in the target population**; this measures the model-based error (epistemic uncertainty), as the difference between an individual’s ‘true model’ prediction ${p}_{i}$ (had the model been derived on perfect information) and estimated prediction ${\widehat{p}}_{i}$ (from the CPM derived with a particular sample size). For example, given a population of size (${n}_{T}$) in the target population, we can define these measures as: the root mean squared prediction error (RMSPE)$=\sqrt{\frac{\sum_{i=1}^{{n}_{T}}{\left({\widehat{p}}_{i}{-p}_{i}\right)}^{2}}{{n}_{T}}}$ the mean absolute prediction error (MAPE)$=\frac{\sum_{i=1}^{{n}_{T}}\left|{\widehat{p}}_{i}{-p}_{i}\right|}{{n}_{T}}$ • **(mis)calibration of predictions in the target population;** calibration measures the agreement between observed and estimated risks, and researchers should quantify the magnitude of any miscalibration. In particular, fitting a logistic regression model in the target population comparing the observed outcome event (${y}_{i}=$0 (no event) or 1 (event)) to the CPM’s estimated predictions${(\widehat{p}}_{i}$) on the log-odds (i.e., logit) scale, then $$\\\begin{array}{l}y_i\sim \mathrm{Bernoulli}\left({p}_{i}\right)\\\mathrm{logit}\left({p}_{i}\right) = {\alpha }_{cal} + {\beta }_{cal}\mathrm{logit}({\widehat{p}}_{i})\end{array}$$ and ${\beta }_{cal}$ is the calibration slope (ideal value of 1) and ${\alpha }_{cal}|{\beta }_{cal}=1$ is the calibration-in-the-large. Moreover, calibration can be assessed graphically using a calibration plot that compares observed and predicted values via a flexible (non-linear) calibration curve fitted using a smoother or splines • **uncertainty in the model’s predictions**; for example, as measured by the width of 95% intervals around the calibration curve; the width of 95% (credible) intervals for each individual’s risk from their posterior distribution, or the effective sample size for each individual’s prediction **Targeting expected values in the population:** For each measure, we might consider the sample size needed to target a particular *expected value*. That is, to seek the sample size so that the CPM is expected to have (i.e., on average) a chosen value (e.g., target a particular mean reduction in c-statistic, mean calibration slope, mean 95% interval width for individual risk or calibration curve) when applied in the target population **Targeting assurance:** Alongside the expected value, the variability (e.g., standard deviation of the reduction in c-statistic, instability of the calibration curve) of values may also be of interest because smaller variability gives more assurance that a single dataset of that size will lead to a reliable CPM. This assurance can be formally quantified as a probability that a particular performance is achieved in the target population. For example, we might consider the sample size needed to develop a CPM that meets the following assurance criteria in the target population: • a probability of > 90% that the calibration slope is between 0.9 and 1.1 • a probability of > 90% that the MAPE or RMSPE is < 0.05 • a probability of > 90% that the reduction in c-statistic is < 0.025

**Table 2:** Classification performance measures of interest for examining expected model degradation and assurance probabilities when using a particular sample size for CPM development

In situations where a risk threshold is of interest for clinical decision making (e.g., to decide when to initiate treatment, refer for biopsy, etc.), then other measures linked to classification are also relevant for quantifying model degradation, including: • **expected probability of misclassification (P(misclassification))**; that is, the proportion of an individual’s posterior distribution that falls on the opposite side of the risk threshold compared to their ‘true’ prediction [15]. • **expected reduction of clinical utility in the target population**, for example as measured by the reduction in net benefit. This is outlined by Sadatsafavi et al., [6] who highlight that the ‘true’ model (i.e., knowing each individual’s true risk, ${p}_{i}$, based on the chosen set of predictors) provides a maximum net benefit ${(NB}_{max}$) that can be achieved (also known as ‘expected value of perfect information' (EPVI)). Then, for a model estimated on a particular sample size, the net benefit of that model (${NB}_{model}$) will be lower than ${NB}_{max}$ due to misclassification compared to their ‘true’ prediction (see previous bullet point); when the probability of misclassification increases due to larger sampling error (lower sample size), the reduction in net benefit will increase. The mean difference in the fitted model’s net benefit and ${NB}_{max}$ in the target population, provides the model’s expected degradation in clinical utility for a particular sample size. Also known as the model’s ‘expected value of sample information’ (EVSI). Alongside this measure of absolute degradation, the *relative* amount of degradation can be summarised as $100\times {(NB}_{model}/{NB}_{max})$ and taking the mean gives the ‘expected relative value of sample information’ (ERVSI) for the model’s net benefit. The value of sampling information might also account for other strategies such as ‘treat none’ and ‘treat all’ (or other models), alongside the model of interest. In particular, based on observed results in the development sample, the strategy with the highest observed net benefit would be the one selected as the ‘winner’. This can vary across samples by chance, and so the true value of sample information is how the winner’s net benefit ${(NB}_{winner})$ degrades compared to that of the true model **Targeting assurance:** Alongside the *expected* value of sample information, the *variability* may also be of interest because smaller variability gives more assurance that a single dataset of that size will be closer to the expected value. This assurance can be formally quantified as a targeted probability; for example: • A probability of 90% that a randomly selected individual has P(misclassification) < 5% • A probability of 90% that ${RVSI}_{model}\ge$90% • A probability of 90% that ${RVSI}_{winner}\ge$90%	

Researchers may identify the (minimum) sample size needed to target a (maximum) acceptable value for the *expected* degradation for each performance measure of interest; that is, what degradation is anticipated on average when developing the model with a particular sample size. For example, they might identify a minimum sample size that targets maximum expected values for the degradation in the c-statistic, the net benefit, and the expected uncertainty (e.g., mean width of 95% uncertainty intervals) around risk estimates.

Rather than targeting *expected* values, an alternative is to target *assurance *defined by a large probability of meeting desired performance criteria. Assurance probabilities are well-known for trial design [17], but here we propose them for model development studies. For example, researchers might consider sample sizes to ensure a probability $\ge$ 0.9 that a developed CPM will have a calibration slope between 0.9 to 1.1 in the target population; a probability $\ge$ 0.9 that the degradation in c-statistic will be < 0.025; or a probability of $\ge$ 0.9 that the degradation in net benefit will be $\le$ 10%.

Those designing a CPM development study must prioritise which performance measures (e.g., calibration, discrimination and net benefit; prediction and classification instability) and target metrics (e.g., expected values, assurance probabilities) are most relevant for their setting and purpose; this requires a multidisciplinary consultation including clinicians and patients. Crucially, any measure of interest can be derived directly from posterior distributions of individuals’ predictions; hence, why the forthcoming sample size calculation needs to anticipate them.

(c) The calculation should be compatible with the planned analysis approach

Posterior distributions for individual-level predictions will depend on the modelling approach used to develop the CPM, and so any *general* sample size calculation needs to be flexible enough to allow *any* modelling approach to be included (e.g., regression, random forests, neural networks). In Section A general simulation-based approach for examining required sample size, we propose a simulation-based approach for this. However, for some modelling options like regression, the anticipated posterior distributions can be closely approximated using statistical theory to allow a faster (computationally less-demanding) approach. In Section An approximation for unpenalised and penalised regression we consider this for (Bayesian penalised) logistic regression.

### A general simulation-based approach for examining required sample size

We now introduce a general framework for calculating and examining the sample size required for CPM development studies, building on the previous section and integrating various statistical concepts, approaches and metrics. The premise is: (i) anticipating the posterior distribution of individual-level predictions that would arise when developing a CPM using a chosen modelling approach with a particular sample size; and subsequently (ii) obtaining and summarising the posterior distributions of this CPM’s predictions and performance in the target population (e.g., using the measures of performance and degradation described in Tables 1 and 2).

As there are a wide variety of model development (supervised learning) approaches of interest for CPM researchers, and closed-form (parametric) approximations for posterior distributions are challenging to anticipate for some approaches (e.g., random forests or deep learning), our general approach uses simulation. Simulation is not a new idea for sample size calculations [18]; notably, Harrell’s textbook illustrates how to use simulation for examining the minimum sample size required to target a particular MAPE [19], assuming a true model with one continuous predictor. Below, we generalise this to include multiple predictors and provide ten steps for how to sample from posterior distributions to summarise the CPM’s anticipated performance and degradation in the target population, compared to an assumed ‘true model’. Here onwards, we refer to this ‘true model’ as a *reference model*, which is a working model (e.g., based on previous models in the field) specified by the user to implement our approach. Example code in Stata and R is available at https://github.com/Richard-D-Riley/code and we intend to embed the approach with our *pmsampsize* package in R, Stata and Python.

#### PART A: Set-up phase to produce datasets for development and testing in the target population

- Step (1) – specify the candidate predictors and any variables linked to fairness checks

All candidate predictors that will be used to develop the CPM need to be specified. This should include *core predictors* that are well-established as important predictors of the outcome, and any exploratory candidate predictors (e.g., additional biomarkers). Also, variables linked to fairness checks should be specified, even if they are not candidate predictors (e.g., those representing protected characteristics and subgroups). Note that, if planning to use an existing dataset for CPM development, only the predictors and variables in that dataset can be chosen.

- Step (2) – specify the joint distribution of the predictors and variables from step (1)

The joint distribution of predictors and variables defines the case-mix of the target population of interest. This can be based on that observed in an existing dataset or a pilot study in the same target population. Sometimes, the development dataset may exist but is unavailable. In this instance, the data holders could be asked to provide a synthetic dataset that mimics the joint distribution, for instance using a simulation-based approach that models conditional relationships [20], such as via *synthpop *in R [21]. For example, Clinical Practice Research Datalink (CPRD) generates synthetic data to improve workflows (https://www.cprd.com/synthetic-data). Sometimes there will be no available data and no joint distribution information; in this case it may be necessary to assume conditionally independent distributions for the predictors. Previous work suggests this can be a reasonable approximation when focusing on some measures of model degradation such as expected calibration slope and MAPE [7, 8]. Different plausible assumptions of the joint distribution can be tried to explore the impact on sample size.

- Step (3) – specify a reference model

To examine the impact of sample size on model degradation, a crucial step is to specify a reference model that expresses (a transformation of) the outcome as a function of assumed effects of the predictors from step (2). Like any sample size calculation, this requires drawing on previous evidence and judgement, for example from existing studies, previous models in the field and clinical expertise. Even if a ‘black box’ CPM development approach (e.g., random forests, neural networks) is planned, we recommend specifying the reference model as a regression equation, as it is pragmatic and interpretable. For example, a logistic regression model could be specified as,

1 $$\\\begin{array}{l}y_i\sim\mathrm{Bernoulli}\left(p_i\right)\\\mathrm{logit}\left(p_{\mathrm i}\right)=\alpha\;+\;\delta\left(\beta_1x_{1i}+\beta_2x_{2\mathrm i}+\cdots+\beta_Px_{Pi}\right)\end{array}$$

with the user defining the $$P$$ parameter values to match anticipated relative predictor weights and model performance in the target population. The $$\beta$$ coefficients provide relative weights of predictors ($$x)$$ on the logit (i.e., log odds) scale. In the absence of other information, and assuming all candidate predictors will add predictive information, each predictor might be standardised and all $$\beta$$ values set to 1. Then, if the user provides the anticipated c-statistic and overall risk, the iterative process of Austin [22] can be used to identify values of $$\alpha$$ and $$\delta$$ that match these (within a small margin of error) in the target population defined by step (2). This process is implemented in our *pmstabilityss* package for R and Stata (available from https://github.com/JoieEnsor) [23]. Note that, to allow for added complexity that machine learning approaches aim to model, the regression model could include additional parameters with interactions, categorised continuous predictors, and non-linear relationships.

When users are planning to consider candidate predictors for which little or no evidence exists about their (added) predictive value, it is conservative to exclude them from the reference model (i.e., assume they are noise variables).

- Step (4) – generate a large dataset that represents the target population

To form a large dataset representative of the target population, randomly sample candidate predictor values from the joint distribution (or the available synthetic dataset) specified in step (2) for a large number of hypothetical individuals. Then, use the reference model (from step (3)) to calculate the true risk (i.e., ${p}_{i}$) for each individual conditional on their predictor values. Randomly generate their observed outcome value (i.e. ${y}_{i}$ = 1 if event, ${y}_{i}$ = 0 if no event) from ${y}_{i} \sim \text{ Bernoulli}({p}_{i})$. We suggest the dataset typically contains at least 100,000 observations, with thousands of outcome events. This allows the performance of the models to be estimated with very little error. However, the most appropriate dataset size will depend on the prevalence of the outcome while also considering the additional computational time of a larger dataset. If relevant, users may also generate missing predictor data (under a plausible mechanism and missingness probability) for some individuals in the dataset.

- Step (5) – generate a development dataset of size $n$ and other relevant samples (e.g., tuning datasets)

Follow the same approach as in step (4), to create a development dataset of $n$ individuals containing their (candidate) predictor and outcome values. If using an existing dataset for model development, then $n$ is fixed. Otherwise, when planning prospective data collection, this is the user’s chosen sample size of interest, perhaps stemming from previous sample size recommendations [5]. Sometimes further samples are used toward model building, such as hold-out samples for tuning and recalibration; if so, these should also be randomly generated for a specified size. If relevant, users may also introduce missing data for some individuals in the development dataset, based on a chosen mechanism and missingness probability (which may, or may not, be the same as that for any missing data in the target population).

#### PHASE B: Develop one CPM using the development dataset and quantify performance in the target population dataset

- Step (6) – develop a CPM applying a chosen modelling approach to the development dataset

Use the development dataset of size $n$ from step (5) to build a CPM using the planned model development approach, which can be any predictive AI-based modelling approach (i.e., any supervised learning method that requires a sample of data containing outcome and predictor values to produce it). It is important to ensure any user-specific choices are properly reflected (e.g., the approach to variable selection and hyperparameter tuning; how a model is recalibrated after testing in a hold-out sample; adjustment for optimism via bootstrapping; handling of any missing data; any sampling approaches to handle class imbalance, such as SMOTE and subsequent miscalibration adjustments [24]).

- Step (7) – apply the developed CPM to make predictions for each individual in the target population dataset

Apply the CPM developed in step (6) to each individual in the large target population dataset from step (4) to obtain a prediction (estimated value, ${\widehat{p}}_{i}$) for their risk (${p}_{i}$), conditional on their observed predictor values (i.e.$,x_{1new},x_{2new},\dots,x_{Pnew})$. If missing data exists for any predictors in the target population dataset, then the user’s chosen approach to handling missing predictors at deployment should be included in this step. We do not consider missing data any further in this article.

- Step (8) – quantify the CPM’s predictive performance and degradation in the target population dataset, both overall and in any subgroups linked to fairness checks

Calculate the CPM’s predictive performance in the large target population dataset from step (4), and the degradation in performance compared to the reference model’s performance in the same large dataset. As the target population dataset is huge, any sampling error in performance and degradation estimates should be tiny and so can be ignored. Any performance measures of interest can be chosen by the user, and generally we recommend to at least consider discrimination, calibration, and net benefit (see Tables 1 and 2). These can also be examined within specific subgroups of interest, such as those defined by ethnicity, as part of any fairness checks.

#### PHASE C: Generate and summarise posterior distributions of CPM parameters, predictions, performance and degradation

- Step (9) – repeat steps (5) to (8) many times and summarise the posterior distributions of predictions, predictive performance and model degradation in the target population dataset

Repeating steps (5) to (7) involves repeatedly sampling a development dataset (of the chosen size, $n$) and building a separate CPM to each, using the same development approach each time. This leads to multiple CPMs, essentially obtained by repeatedly sampling from the joint posterior distribution of the model parameters. Generally, we recommend at least 1000 samples to ensure the Monte Carlo (MC) simulation error is relatively small for most measures, although our experience suggests some measures (e.g., net benefit, MAPE) may be quite well estimated even in a lower number of samples (e.g., 200). We refer to Morris et al. for more consideration of simulation size and MC error [25].

Applying each of these (e.g., 1000) CPMs to each individual in the target population dataset generates multiple predictions (i.e. multiple ${\widehat{p}}_{i}$ values) per individual, reflecting their posterior distribution (i.e. $\mathrm{p}({p}_{i} |{\boldsymbol{\upbeta}},{{\boldsymbol{x}}}_{new})$). From this, uncertainty measures can be derived (such as a 95% interval for each individual’s true ${p}_{i}$ derived from 2.5 and 97.5 percentile values of the distribution) and displayed (e.g., using a prediction instability plot comparing each individual’s 1000 risk estimates versus their true risk) [15].

Repeating step (8) many times also produces posterior distributions for measures of CPM performance and degradation in the target population. For example, consider the c-statistic and the calibration slope. In terms of the degradation of the c-statistic we obtain 1000 estimates of:

$${\begin{aligned}\mathrm{model}&\;\mathrm{degradation}\;\mathrm{in}\;\mathrm{c-statistic}\;\\&=\;\mathrm{(c-statistic}\;\mathrm{of}\;\mathrm{developed}\;\mathrm{model})\;-\;\mathrm{(c-statistic}\;\mathrm{of}\;\mathrm{true}\;\mathrm{model})\end{aligned}}$$

For the calibration slope, we obtain 1000 estimates of:

$${\begin{aligned}\mathrm{model}&\;\mathrm{degradation}\;\mathrm{in}\;\mathrm{calibration}\;\mathrm{slope}\;\\&=\;\mathrm{(calibration}\;\mathrm{slope}\;\mathrm{of}\;\mathrm{developed}\;\mathrm{model})\;-\;\mathrm{(calibration}\;\mathrm{slope}\;\mathrm{of}\;\mathrm{true}\;\mathrm{model)}\;\\&=\;\mathrm{(calibration}\;\mathrm{slope}\;\mathrm{of}\;\mathrm{developed}\;\mathrm{model})\;-\;\mathrm{1}\end{aligned}}$$

Each posterior distribution can be summarised, for example in terms of the mean (expected) value, and measures of uncertainty and assurance (e.g., mean and 95% interval for calibration slope; probability that degradation in net benefit will be $\le$ 10%). They can also be displayed, for example, in an instability plot of calibration curves. Again, it is entirely the user’s decision as to which measures to focus on, and we recommend summarising each chosen measure using both their original values and their corresponding degradation values.

#### PHASE D (OPTIONAL): Examine other sample sizes and reference models

- Step (10): Repeat steps (5) to (9) for alternative development sample sizes and assumptions

Unless using an existing dataset with a fixed $n$ for model development, most users will want to examine a range of sample sizes, particularly when planning prospective data collection. In this instance, they will want to identify a (minimum) sample size that is expected to meet some target criteria of their choice (e.g., mean degradation in c-statistic < 0.02; mean degradation in net benefit < 10%; probability $\ge$ 90% that calibration slope $\ge$ 0.9 and $\le 1.1$; probability of 90% that degradation in net benefit $\le$ 10%;). Similarly, they may want to repeat the process with different (yet still plausible) reference models and identify how the required sample size changes.

#### Extension: embedding uncertainty in the reference model within the simulation process

Rather than repeating the whole process with different reference models, the uncertainty in the reference model can be embedded in the simulation itself. That is, the simulation process can randomly select which reference model is used in step (3), from a specified set of plausible reference models. For example, multiple reference models could be specified that encompass a range of c-statistics, and those with the most optimistic c-statistics could be given a lower selection probability than others. More broadly, a distribution of plausible reference model parameters could be specified, to reflect uncertainty in the anticipated outcome prevalence, c-statistic and relative predictor weights. It may be pragmatic to focus on just expressing uncertainty in the c-statistic (and so, keep fixed the prevalence and *relative* predictor weights).

### An approximation for unpenalised and penalised regression

A fully simulation-based approach can be time consuming. To reduce computational burden and carbon footprint, we now utilise a decomposition of Fisher’s information to approximate anticipated posterior distributions for predictions in new individuals ($\mathrm{p}({p}_{i} |{\boldsymbol{\upbeta}},{{\boldsymbol{x}}}_{new}))$ from (penalised) regression via a decomposition of Fisher’s information, extending earlier work [9, 10]. The approach has connections with Approximate Bayesian Computation and generalized Bayesian inference, which aim to approximate or replace the exact likelihood to reduce computational issues, and closely aligns with the Laplace approximation for Bayesian inference based on a multivariate normal asymptotic [26–29].

#### Approximation for unpenalised logistic regression

After fitting an unpenalised logistic regression model using maximum likelihood estimation, the approximate sampling distribution for the parameter estimates ($\widehat{{\boldsymbol{\upbeta}}}={(\widehat{\alpha },\widehat{\beta }}_{1},{\widehat{\beta }}_{2},\dots ,{\widehat{\beta }}_{\mathrm{P}}){\mathbf{^{\prime}}}$) is multivariate normal. If we assume noninformative (i.e., flat improper) prior distributions, we can express the posterior distribution for ${\boldsymbol{\upbeta}}$ (the true but unknown parameters) as approximately:  

2 $${\boldsymbol{\upbeta}}|\mathbf{y},\mathbf{X}\sim MVN\left(\widehat{{\boldsymbol{\upbeta}}}, \mathrm{var}(\widehat{{\boldsymbol{\upbeta}}})\right)$$

where $\mathbf{X}$ is the design matrix formed using each individual’s predictor values corresponding to ${{\boldsymbol{x}}}_{{\boldsymbol{i}}}=\left(1,{x}_{1\mathrm{i}},{x}_{2\mathrm{i}},\dots ,{x}_{\mathrm{Pi}}\right)$, and $\mathbf{y}$ is the individuals’ observed outcome values ($\mathbf{y}\mathbf{^{\prime}}=({y}_{1},{y}_{2}, \dots ,{y}_{n}))$ in the development dataset. This can be re-expressed in terms of sample size and unit information by [9, 10],

3 $${\boldsymbol{\upbeta}}|\mathbf{y},\mathbf{X}\sim MVN\left(\widehat{{\boldsymbol{\upbeta}}}, { n}^{-1}{\mathbf{I}}^{-1}\right)$$

where $\mathbf{I}$ is Fisher’s *unit* information matrix ($\mathbf{I}$) and $n$ the development sample size. The unit information matrix accounts for the case-mix distribution in the development dataset and the linear predictor of the fitted model. For a logistic regression model, $\mathbf{I}$ is defined by,

4 $$\mathbf{I}=E\left(\frac{\mathrm{exp}\left({\mathbf{X}}{\mathbf{^{\prime}}}\widehat{{\boldsymbol{\upbeta}}}\right)}{{\left(1+\mathrm{exp}\left({\mathbf{X}}{\mathbf{^{\prime}}}\widehat{{\boldsymbol{\upbeta}}}\right)\right)}^{2}}{\mathbf{X}}{\mathbf{^{\prime}}}\mathbf{X}\right)$$

and $E$ denotes the expected value of the matrix, and so requires averaging (i.e., obtaining mean values of) each component of the matrix across all individuals in the development dataset.

As it is independent of sample size, $\mathbf{I}$ only needs to be calculated once and can be obtained by averaging across individuals in the real (or synthetic) dataset (from step (2)) at the end of step (4) of the simulation-based approach; assuming estimates are approximately unbiased, $\widehat{{\boldsymbol{\upbeta}}}$ is replaced with the ${\boldsymbol{\upbeta}}$ of the reference model. Crucially, $\mathbf{I}$ is then fixed for subsequent sample size calculations and so the user only needs to specify their sample size of interest ($n$) to produce the anticipated posterior distribution using Eq. (3). This distribution can then be sampled from directly to obtain, say, 1000 models; hence it avoids requiring Steps (5) or (6) of the simulation-based approach, reducing computational time, though serves as an approximation (see limitations below).

#### Approximation for Bayesian penalised logistic regression

To improve computational speed for Bayesian penalised regression model, we propose a Bayesian one-sample analysis that approximates $$\mathrm{p}({\boldsymbol{\upbeta}}|\mathbf{y},\mathbf{X})$$ by combining (i) the data (likelihood) defined by anticipated parameter estimates (and the corresponding information matrix) from an unpenalised regression of a particular sample size ($$n)$$, and (ii) prior distributions that reflect a penalised regression, for example for a lasso or ridge penalty. Specifically, we approximate,

$$\mathrm{p}({\boldsymbol{\upbeta}}|\mathbf{y},\mathbf{X})\propto \mathrm{p}({\boldsymbol{\upbeta}})\mathrm{p}(\mathbf{y}|{\boldsymbol{\upbeta}},\mathbf{X})$$

by replacing the likelihood of $\mathrm{p}(\mathbf{y}|{\boldsymbol{\upbeta}},\mathbf{X})$ with $\mathrm{p}(\widehat{{\boldsymbol{\upbeta}}}|{\boldsymbol{\upbeta}})$, where the latter is assumed $MVN\left({\boldsymbol{\upbeta}}, {n}^{-1}{\mathbf{I}}^{-1}\right)$ based on an *unpenalised* logistic regression, with $n$ a chosen sample size of interest, and $\mathbf{I}$ assumed known and obtained as described in Section Approximation for unpenalised logistic regression. As mentioned, this is akin to the Laplace approximation to Bayesian inference [28, 29]. Crucially, our ‘data’ is now $\widehat{{\boldsymbol{\upbeta}}}$ which is simply a vector set to the ${\boldsymbol{\upbeta}}$ of the reference model. This assumes parameter estimates are unbiased; a reasonable approximation unless effective sample size is small (see limitations below).

The choice of (shrinkage) prior distributions for the model parameters is flexible and a broad introduction is given by van Erp et al. [30] Here, we assume a vague prior distribution for the intercept, $\mathrm p(\upalpha)\sim N(0,1000000)$ and independent prior distributions for the predictor effects, with a penalty parameter $\lambda$ that defines the amount of shrinkage; larger values correspond to larger shrinkage. Taking a fully Bayesian approach, we also specify a prior distribution (a hyperprior) for $\lambda$ to propagate the uncertainty of $\lambda$ which can often be considerable [31]. Any priors could be chosen in principle; here, we use prior distributions that mirror ridge and lasso penalties [32]:

$$\begin{array}{c}\text{Ridge: p}\left(\beta\right)\sim N(0,\lambda^2)\;\;\;\;\\\mathrm{p}\left({\lambda }^{2}\right)\sim \mathrm{inverse}\_\mathrm{gamma}(0.01, 0.01)\\\text{Lasso: p}\left(\beta \right)\sim \mathrm{laplace}(0, \sqrt{{\lambda }^{-2}})\;\;\;\;\;\;\;\;\\\mathrm{p}\left({\lambda }^{2}\right)\sim \mathrm{gamma}(1,\frac{1}{1.78})\end{array}$$

The term $\mathrm{p}\left(\beta \right)$ refers to the same prior distribution for any of the $\beta$ terms in the model (i.e., ${\beta }_{1}, {\beta }_{2},\dots ,{\beta }_{\mathrm{P}}$). We fit the Bayesian model using MCMC estimation via the Metropolis–Hastings approach, with a burn-in of 10,000 and subsequently take 1000 samples (to mirror the 1000 models generated in the simulation-based approach), with thinning to reduce any autocorrelation by taking every 10th sample until 1000 are obtained overall. Example code is provided at https://github.com/Richard-D-Riley/code using the package *bayesmh* in Stata.

Once the posterior distribution is derived for a chosen sample size, we can sample β values and use them to make predictions for each individual in the large target population dataset. This leads to, say, 1000 risk estimates for each individual (i.e., 1000 samples from their posterior distribution ($\mathrm{p}({p}_{i} |{\boldsymbol{\upbeta}},{{\boldsymbol{x}}}_{new}))$), which are then used in step (9) to derive posterior distributions for the model’s performance and degradation in the target population.

#### Limitations with using an approximation based on Fisher’s decomposition

The approach based on decomposing Fisher’s information substantially improves computational time (see Section Computational time), but it is an approximation based on asymptotic (large-sample) maximum likelihood theory. Hence, it will perform best when the effective sample size is not small (in terms of number of participants and number of outcome events) and in low dimensional settings (i.e., not when the number of predictor parameters is large relative to the number of events). Section Computational time demonstrates this with some examples. In general, we recommend the approximation as a way to more quickly help researchers examine multiple sample sizes and settings of interest; then, a few specific sample sizes and settings can be checked using the full simulation-based approach.

## Results

Now we illustrate the approaches by considering a CPM for the risk of adverse outcomes (by hospital discharge) in pregnant women diagnosed with pre-eclampsia, which is an area of active research. We build on Thangaratinam et al. as a foundation, [33, 34] which reports a penalised regression model based on 9 predictors and an overall outcome risk of 0.68. The question is: what (minimum) sample size is needed to reliably build a new model with these nine predictors (and potentially others), that extends (updates) previous models in this field?

### Applied example: simulation set-up, model development approaches and performance measures

We apply the fully simulation-based sample size calculation of Section A general simulation-based approach for examining required sample size, for each of the following development methods separately: an unpenalised logistic regression (no variable selection), an unpenalised logistic regression followed by a heuristic uniform shrinkage [35], penalised logistic regression with either a ridge or lasso penalty in both frequentist (using ten-fold cross validation for tuning) [36, 37] and Bayesian frameworks (using the prior distributions of Section Approximation for Bayesian penalised logistic regression); a random forest with 100 trees and depth of either 3 (low) or 15 (high) for contrast; and gradient boosting of ensemble decision trees for binary classification, with 100 trees and depth of 15 via the H2O module in Stata [38]. For brevity, a summary of our steps is presented in Table 3. Stata and R code for evaluating the sample size required for the various model development strategies is available at https://github.com/Richard-D-Riley/code. The reference model included 10 parameters for the 9 predictors, with a corresponding c-statistic of 0.76 and a net benefit of 0.41 at a risk threshold of 0.5 suggested by Thangaratinam et al. for decision making (e.g., transfer to tertiary units if risk $\ge$0.5) [34].

**Table 3:** The 10-step process of the fully simulation-based sample size calculation, as applied to a new study aiming to develop a prediction model for the risk of adverse outcomes (by discharge) in pregnant women with pre-eclampsia

**Step (1)—specify the candidate predictors and any variables linked to fairness checks** Building on Thangaratinam et al., [33, 34] we focus initially on nine predictors of: maternal age at pregnancy (years), gestational age at diagnosis (weeks), medical history (i.e., none, 1, or 2 + conditions), urine protein creatinine ratio (mg/mmol), serum urea (mmol/L), serum creatinine (µmol/L), systolic blood pressure (mmHg), parenteral anti-hypertensive therapy, and parenteral magnesium sulphate administered before or within 24 h of diagnosis **Step (2) – specify the joint distribution of the predictors and variables from step (1)** The original authors provided a large (~ 1.6 million) synthetic dataset, generated via *synthpop *in R [21], that closely reflected their original dataset in terms of the joint distribution of predictors, with an overall outcome incidence (risk) of 0.68 **Step (3)—specify a reference model** Based on previous literature, we specified the following reference model with nine predictors (10 parameters): $$\mathrm{logit}\left({p}_{i}\right)= 14.8246-\left(0.204\times \mathrm{age}\right)-\left(5.2265\times \mathrm{ln}\left(\text{gestational age}\right)\right)-\left(0.3243\times \left(1\text{ previous condition}\right)\right)-\left(0.6236\times \left(\ge 2\text{ previous conditions}\right)\right)+\left(0.1665\times \mathrm{ln}\left(\text{creatinine ratio}\right)\right)+\left(0.4574\times \mathrm{ln}\left(\mathrm{urea}\right)\right)-$$$$\left(0.0038\times \mathrm{creatinine}\right)+\left(0.0232\times \text{systolic blood pressure}\right)+\left(0.4552\times \text{antihypertensive treatment}\right)+(1.1425\times \text{magnesium sulphate treatment})$$ In the target population dataset (see below) this reference model has a c-statistic of 0.76, a calibration slope of 1 and a net benefit of 0.41 at a risk threshold of 0.5 considered relevant for decisions **Step (4) – generate a large dataset that represents the target population** We took a random sample of 100,000 individuals from the provided synthetic dataset and reserved these as our target population dataset. We randomly generated each individual’s observed outcome value (i.e. $${y}_{i}$$= 1 if event, $${y}_{i}$$ = 0 if no event) as $${y}_{i} \sim \text{ Bernoulli}\left({p}_{i}\right)$$, with $${p}_{i}$$ defined by the reference model of Step (3) **Step (5) – generate a development dataset of size **$${\boldsymbol{n}}$$** and other relevant samples (e.g., tuning datasets)** We took a random sample of size $$n$$ from the ~ 1.5million individuals that remained in the synthetic dataset (i.e. those not moved to the target population dataset) and randomly generated their observed outcome value as $${y}_{i} \sim \text{ Bernoulli}\left({p}_{i}\right)$$, where $${p}_{i}$$ was defined by the reference model of Step (3). We initially consider $$n$$ = 456, the minimum sample size required to target a calibration slope of 0.9, as calculated using pmsampsize **Step (6) – develop a CPM applying a chosen modelling approach to the development dataset** We consider various approaches for fitting the CPM: unpenalised logistic regression with and without subsequent uniform shrinkage; logistic regression with either a ridge or lasso penalty estimated in either a frequentist or Bayesian framework; random forests, and gradient boosting. For the ridge and lasso approaches, ten-fold cross-validation was used to estimate the tuning parameter (lambda) when using a frequentist framework, and in the Bayesian framework the penalty was induced by the chosen priors shown in Section Approximation for Bayesian penalised logistic regression, with thinning and a burn-in of 5000 samples. When fitting all the models, we standardised predictors **Step (7) – apply the developed CPM to make predictions for each individual in the target population dataset** For each CPM developed in step (6), we applied the fitted model to estimate risk for each of the *N* individuals **Step (8) – quantify the CPM’s predictive performance and degradation in the target population dataset, both overall and in any subgroups linked to fairness checks** We examine (degradation in) each model’s performance in terms of discrimination (c-statistic), calibration (calibration slope and curve) and clinical utility (net benefit at a risk threshold of 0.5), as all these were deemed important and relevant for either patients, clinicians or methodologists **Step (9) – repeat steps (5) to (8) many and summarise the sampling (posterior) distributions of predictions, predictive performance and model degradation using the target population dataset** We repeated steps (5) to (8) 999 times; then for each CPM approach, we used the 1000 sets of performance results to summarise (degradation) in discrimination, calibration and clinical utility. We also produced calibration, prediction and classification instability plots [15]. **Step (10): Repeat steps (5) to (9) for alternative development sample sizes and assumptions** We repeated the whole process considering a range of other sample sizes; and the impact of an additional 10 candidate continuous predictors (all standardised and independent) that are noise variables	

Our focus is on the anticipated (degradation in) CPM performance in terms of discrimination (c-statistic), calibration (calibration slope and curve) and clinical utility (net benefit at a risk threshold of 0.5); and the MAPE and 95% interval width around individual risk estimates. For brevity, we focus our evaluations on three sample sizes: $n$ = 75, 335 and 456. A sample size of $n$ = 456 (about 310 events and 31 events per candidate predictor parameter for regression models) is the *minimum* recommended by *pmsampsize* [5, 39, 40], that targets a calibration slope of 0.9 based on a closed-form (heuristic) solution for the uniform shrinkage required for unpenalised logistic regression, assuming the c-statistic is 0.76 and overall risk is 0.68. A sample size of $n$ = 335 (about 241 events and 24 events per candidate predictor parameter) is that recommended for precise estimation of the overall risk (target 95% CI width of 0.1, assuming overall risk is 0.68); and $n$ = 75 provides a much smaller development dataset for comparison (about 51 events and 5 events per candidate predictor parameter).

### Applied example: results and key learning points for users of the framework

**Fig. 1:**
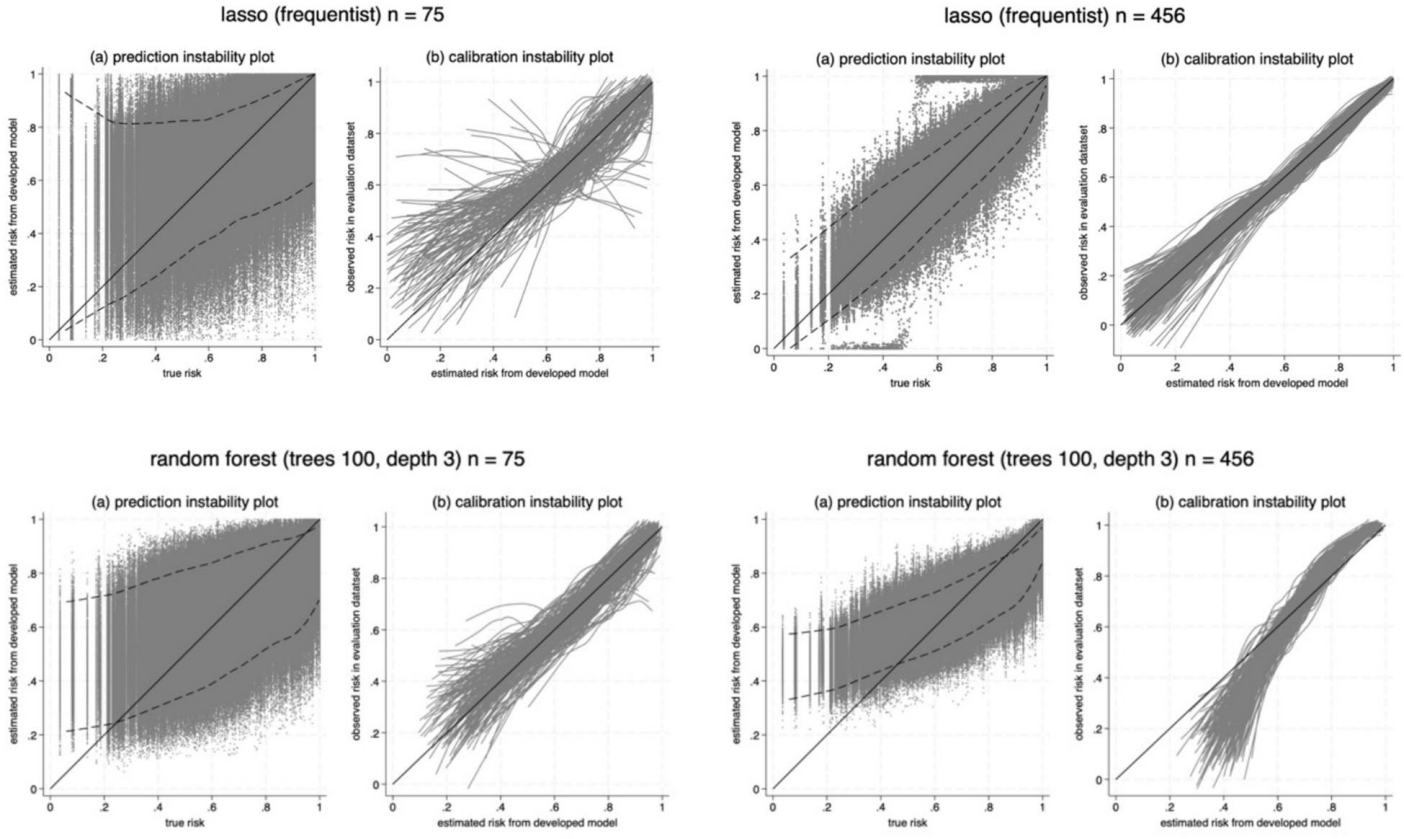
Prediction and calibration instability plot, showing anticipated uncertainty of individual risk estimates and calibration curves in a large evaluation dataset (the target population), when developing a CPM using a development sample of *n* = 75 or *n* = 456 and a modelling strategy of either: logistic regression with a lasso penalty, or random forests using 100 trees with depth 3. For ease of display, only 200 calibration curves are presented

**Fig. 2:**
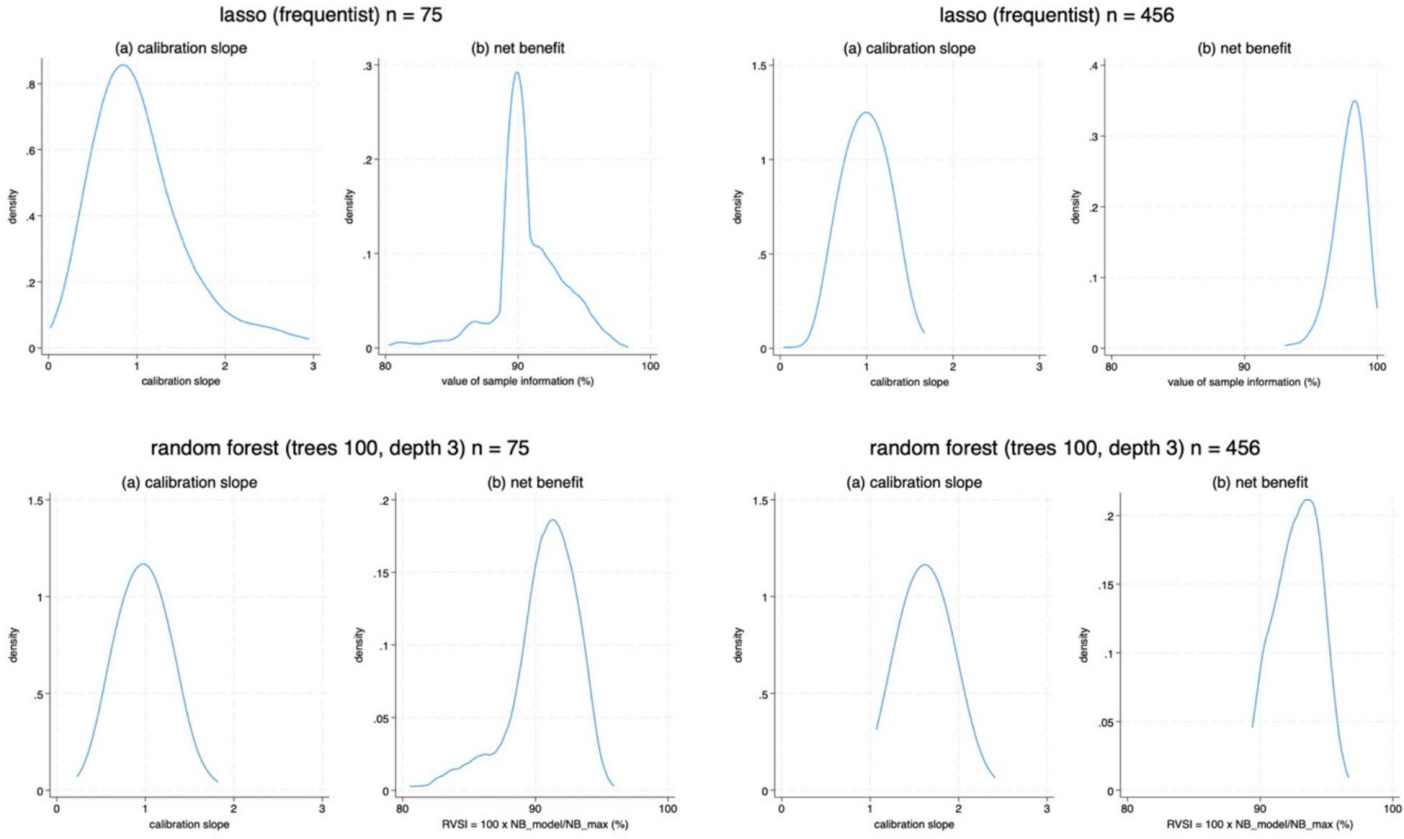
Anticipated posterior distribution of calibration slope and value of information (here, the RVSI: the relative value of sample information) for the fitted model’s net benefit compared to the reference model in a large evaluation dataset (target population), when using a development sample of *n* = 75 or *n* = 456 and a model development strategy of either: logistic regression with a lasso penalty, or random forests using 100 trees with depth 3

Table 4, Table S1 and Table 5 provide results for the pre-eclampsia model using development sample sizes of $n$ = 75, 355 and 456, respectively. They summarise anticipated model performance, degradation and instability for various modelling strategies. Figures 1 and 2 provide graphical displays of the posterior distributions for a few of the modelling strategies and performance metrics. We now summarise the results as a series of key learning points, intended to help inform future users of our proposed framework about the consequence of small sample sizes, the impact of different modelling choices, and how the framework embeds and generalises previous sample size calculations. Note that, in this particular application the net benefit results for the model were very similar to those of the winner (as defined in Table 2), so we only discuss the former here.

**Table 4:** Summary of posterior distributions (based on 1000 samples) of the anticipated CPM performance, degradation and instability in a large evaluation dataset, for CPMs developed with particular modelling approaches using a sample size of 75 participants (~ 51 events) and 10 predictor parameters. Degradation is examined relative to the performance of the reference model shown in Table 3, which has a calibration slope of 1, c-statistic of 0.76, and net benefit of 0.41 in the target population. ERVSI was very similar for the winning strategy

		***Error and uncertainty of predictions from CPM***		***Calibration, discrimination and clinical utility of CPM***		
**CPM development approach**	**Sample size approach**	**MAPE:** mean (95% range)	**95% interval width:** mean (95% range)	**Calibration slope:** mean (95% range) P(0.9 < slope < 1.1) P(0.85 < slope < 1.15)	**C-statistic:** mean (95% range) mean degradation (95% range)	**Net benefit:** mean (95% range) ERVSI (95% RVSI range) P(RVSI > 90%)
Unpenalised logistic regression (frequentist)	Fully simulation-based	0.15 (0.09 to 0.22)	0.66 (0.26 to 0.94)	0.45 (0.17 to 0.83) 0.01 0.02	0.68 (0.60 to 0.73) −0.08 (−0.16 to −0.03)	0.36 (0.30 to 0.39) 87.8% (73.9% to 96.4%) 0.42
Unpenalised logistic regression + heuristic shrinkage	Fully simulation-based	0.13 (0.07 to 0.20)	0.54 (0.35 to 0.81)	1.17 (−5.06 to 7.58) 0.11 0.18	0.67 (0.33 to 0.74) −0.09 (−0.43 to −0.03)	0.37 (0.32 to 0.39) 90.3% (79.6% to 96.3%) 0.56
Ridge logistic regression (frequentist)	Fully simulation-based	0.11 (0.06 to 0.16)	0.42 (0.29 to 0.67)	1.80 (0.34 to 7.15) 0.15 0.21	0.70 (0.61 to 0.74) −0.06 (−0.15 to −0.02)	0.37 (0.35 to 0.40) 92.5% (85.6% to 97.7%) 0.79
Ridge logistic regression (Bayesian)	Fully simulation-based	0.11 (0.06 to 0.17)	0.48 (0.28 to 0.76)	1.27 (0.35 to 4.02) 0.12 0.20	0.70 (0.62 to 0.70) −0.06 (−0.14 to −0.02)	0.37 (0.34 to 0.39) 91.9% (82.9% to 97.6%) 0.75
Lasso logistic regression (frequentist)	Fully simulation-based	0.13 (0.07 to 0.18)	0.47 (0.32 to 0.71)	0.95 (0 to 3.18) 0.13 0.18	0.65 (0.5 to 0.73) −0.11 (−0.26 to −0.03)	0.37 (0.33 to 0.39) 90.4% (82.3% to 95.4%) 0.49
Lasso logistic regression (Bayesian)	Fully simulation-based	0.13 (0.07 to 0.19)	0.59 (0.16 to 0.90)	0.53 (0.25 to 0.94) 0.03 0.04	0.69 (0.61 to 0.74) −0.07 (−0.15 to −0.02)	0.37 (0.32 to 0.39) 90.8% (80.5% to 97.5%) 0.65
Random forest (100 trees, depth 3)	Fully simulation-based	0.12 (0.09 to 0.16)	0.42 (0.27 to 0.50)	0.97 (0.60 to 1.42) 0.40 0.57	0.68 (0.61 to 0.71) −0.08 (−0.15 to −0.05)	0.37 (0.34 to 0.38) 90.6% (83.2% to 94.4%) 0.69
Random forest (100 trees, depth 15)	Fully simulation-based	0.14 (0.10 to 0.18)	0.57 (0.33 to 0.70)	0.56 (0.33 to 0.75) 0 0	0.66 (0.58 to 0.70) −0.10 (−0.17 to −0.06)	0.36 (0.32 to 0.38) 87.5% (77.7% to 93.1%) 0.29
Gradient boosting (100 trees, depth 15)	Fully simulation-based	0.24 (0.21 to 0.28)	0.88 (0.36 to 0.99)	0.18 (0.07 to 0.24) 0 0	0.64 (0.55 to 0.68) −0.12 (−0.21 to −0.08)	0.33 (0.28 to 0.37) 81.2% (68.7% to 90.0%) 0.02

**Table 5:** Summary of posterior distributions (based on 1000 samples) of the anticipated CPM performance, degradation and instability in a large evaluation dataset, for CPMs developed with particular modelling approaches using a sample size of 456 participants (~ 310 events) and 10 predictor parameters. Degradation is examined relative to the performance of the reference model shown in Table 3, which has a calibration slope of 1, c-statistic of 0.76, and net benefit of 0.41 in the target population. ERVSI was very similar for the winning strategy

		***Error and uncertainty of predictions from CPM***		***Calibration, discrimination and clinical utility of CPM***		
**CPM development approach**	**Sample size approach**	**MAPE:** mean (95% range)	**95% interval width:** mean (95% range)	**Calibration slope:** mean (95% range) P(0.9 < slope < 1.1) P(0.85 < slope < 1.15)	**C-statistic:** mean (95% range) mean degradation (95% range)	**Net benefit:** mean (95% range) ERVSI (95% RVSI range) P(RVSI > 90%)
Unpenalised logistic regression (frequentist)	Fully simulation-based	0.051 (0.030 to 0.075)	0.25 (0.05 to 0.50)	0.89 (0.69 to 1.14) 0.36 0.57	0.75 (0.73 to 0.76) −0.01 (−0.03 to 0)	0.40 (0.39 to 0.40) 97.9% (95.5% to 99.6%) 1.0
Unpenalised logistic regression + heuristic shrinkage	Fully simulation-based	0.050 (0.030 to 0.073)	0.24 (0.07 to 0.42)	0.98 (0.74 to 1.32) 0.46 0.66	0.74 (0.73 to 0.75) −0.02 (−0.03 to −0.01)	0.40 (0.39 to 0.40) 98.0% (95.5% to 99.6%) 1.0
Ridge logistic regression (frequentist)	Fully simulation-based	0.048 (0.028 to 0.071)	0.22 (0.07 to 0.38)	1.10 (0.82 to 1.50) 0.45 0.62	0.74 (0.73 to 0.75) −0.02 (−0.03 to −0.01)	0.40 (0.39 to 0.40) 98.1% (95.6% to 99.6%) 1.0
Ridge logistic regression (Bayesian)	Fully simulation-based	0.050 (0.029 to 0.073)	0.24 (0.07 to 0.43)	1.00 (0.74 to 1.35) 0.50 0.70	0.74 (0.73 to 0.75) −0.02 (−0.03 to −0.01)	0.40 (0.39 to 0.40) 98.0% (95.9% to 99.6%) 1.0
Lasso logistic regression (frequentist)	Fully simulation-based	0.051 (0.030 to 0.074)	0.24 (0.07 to 0.43)	0.99 (0.76 to 1.33) 0.53 0.75	0.74 (0.73 to 0.75) −0.02 (−0.01 to −0.03)	0.40 (0.38 to 0.40) 97.6% (94.5% to 99.2%) 1.0
Lasso logistic regression (Bayesian)	Fully simulation-based	0.052 (0.031 to 0.077)	0.25 (0.06 to 0.47)	0.89 (0.67 to 1.16) 0.39 0.60	0.74 (0.73 to 0.75) −0.02 (−0.03 to −0.01)	0.40 (0.38 to 0.40) 97.9% (94.8% to 99.5%) 1.0
Random forest (100 trees, depth 3)	Fully simulation-based	0.090 (0.079 to 0.102)	0.21 (0.14 to 0.27)	1.63 (1.26 to 2.09) 0 0	0.72 (0.71 to 0.73) −0.04 (−0.05 to −0.03)	0.38 (0.36 to 0.39) 92.8% (89.7% to 95.6%) 0.95
Random forest (100 trees, depth 15)	Fully simulation-based	0.112 (0.099 to 0.127)	0.52 (0.20 to 0.72)	0.62 (0.54 to 0.71) 0 0	0.69 (0.67 to 0.70) −0.07 (−0.09 to −0.06)	0.37 (0.36 to 0.38) 90.7% (87.7% to 93.2%) 0.70
Gradient boosting (100 trees, depth 15)	Fully simulation-based	0.22 (0.20 to 0.23)	0.88 (0.09 to 0.98)	0.22 (0.19 to 0.25) 0 0	0.67 (0.65 to 0.69) −0.09 (−0.11 to −0.07)	0.35 (0.34 to 0.37) 86.4% (82.5% to 89.9%) 0.02

#### Model degradation increases as sample size decreases

Regardless of the model development strategy, smaller sample sizes lead to CPMs with lower expected performance (higher expected model degradation), which agrees with recent research [2]. For example, consider the Bayesian lasso regression. The model’s expected c-statistic is 0.69 at $n$ = 75 and 0.74 at $n$ = 456, corresponding to an expected degradation in the c-statistic of 0.07 and 0.02, respectively. In terms of net benefit, the model’s expected *relative* value of sample information (ERVSI = $100\times {(NB}_{model}/{NB}_{max})$) is 90% at $n$ = 75 and 97% at $n$ = 456. Lowering the sample size can also adversely impact the expected calibration slope, even when using penalisation or ensemble methods that aim to address overfitting. For example, when using a frequentist ridge regression to develop the CPM, the expected calibration slope is 1.80 at $n$ = 75 and 1.10 at $n$ = 456.

#### Variability in performance and degradation increases as sample size decreases

For all development strategies, variability in CPM performance increases as sample size decreases. This is important because, even if the expected value is acceptable (e.g., calibration slope of 1), larger variability reduces assurance that the CPM will perform well in one particular realisation of a development dataset. For example, when using the frequentist lasso approach with $n$ = 75, the CPM has an expected calibration slope of 0.95, and thus quite close to 1. However, the posterior distribution for the calibration slope has a 95% range from 0 to 3.18 (Fig. 2), indicating potentially large miscalibration in any one particular dataset. In contrast, with $n$ = 456, the expected value is 0.99 (thus, still close to 1) and the 95% range of 0.76 to 1.33 is much narrower (though still quite wide). The distribution of anticipated calibration curves is thus much narrower for an $n$ of 456 compared to 75 (Fig. 1).

#### Assurance decreases as sample size decreases

A major consequence of the first two points is that assurance probabilities reduce as sample size decreases. For example, the tables show lower assurance probabilities for calibration slope and RVSI when $n$ is 75 compared to 456. For instance, for a CPM developed using logistic regression with a uniform (heuristic) shrinkage factor, the probability that RVSI $\ge$ 90% (i.e., the proportion of the RVSI posterior distribution with values $\ge$ 90%) is 1 when $n$ is 456 but 0.55 when $n$ is 75. Similarly, the probability(0.9 $\le$ calibration slope $\le$ 1.1) is 0.46 when $n$ is 456 but drops to 0.11 when $n$ is 75.

#### The framework embeds and extends existing sample size criteria

Recall, previous sample size approaches provide closed-form solutions to target expected values, such as a calibration slope of 0.9 for logistic regression or a MAPE of 0.05. These targets can still be considered in our approach; for example, for an $n$ of 456, Table 5 shows that the expected calibration slope for an unpenalised logistic regression is 0.89, close to the 0.9 value that Riley’s closed-form solution anticipates. However, the new approach enables *any* performance and degradation metric of interest to be summarised, and reveals variability and assurance probabilities, in addition to expected values, which may strongly influence the final sample size desired.

#### Addressing individual-level stability and fairness may require the largest sample sizes

Following the previous point, a larger sample size is needed to target sufficient stability of risk estimates at the individual-level and for all subgroups. For example, Fig. 1 shows prediction instability plots for lasso and random forest models, which reveal large uncertainty around individual risk estimates (even spanning about 0 to 1 for some individuals), even at an $n$ of 456 where calibration curves appear quite stable. The magnitude of uncertainty may also be quite discrepant across relevant subgroups (e.g., defined by ethnicity), and larger sample sizes may be needed to address this [9, 41].

#### The sample size required depends on the model development approach

The approach used for CPM development also impacts the amount of degradation and the assurance probabilities. For example, with an $n$ of 456, the probability(0.9 $\le$ calibration slope $\le$ 1.1) is 0.70 for a Bayesian ridge regression, 0.45 for a frequentist ridge regression, and 0 for a random forest or gradient boosting model (Table 5). Model tuning will also impact the required sample size. Tables 4 and 5 show that performance degradation and assurance probabilities are worse when using a random forest with a tree depth of 15 compared to 3. With an $n$ of 456, the expected c-statistics are 0.72 and 0.69, and the mean uncertainty interval widths for individual risk are 0.21 and 0.52, for tree depths of 3 and 15, respectively. Gradient boosting appears to have the largest degradation in this particular example, with lowest expected degradation in net benefit for the sample sizes considered.

#### Context and threshold choice is important

The EVSI and ERVSI for net benefit is high, even at a small $n$ of 75, but this also reflects the threshold choice (0.5) lower than the overall prevalence (0.68). Even if a small sample leads to, say, a lasso model shrinking all predictor effects close to zero, the final model gives all individuals a risk estimate of about 0.68 plus sampling error; thus, most individuals will be above the threshold. This is akin to the fitted model becoming resembling the ‘treat all’ strategy, which has a net benefit of 0.36 in the evaluation dataset (88% of the reference model’s net benefit); hence the fitted model’s RVSI is inevitably going to be high. Had the threshold choice been, say, 0.9 then a highly penalised model would class most individuals to be below the threshold, and this more closely reflects the ‘treat none’ strategy, and so the model’s RVSI would be closer to 0%.

#### Adding candidate predictors and noise variables increase the sample size required

The impact of a larger tree depth also illustrates a broader point: as model complexity increases, the potential for model degradation and instability increases. This also occurs when adding in additional candidate predictors, especially if many of those are noise variables. Table S2 shows the impact of adding 10 extra candidate predictors, which are all considered to be noise variables, which is a conservative assumption (e.g., when researchers are exploring whether additional variables improve CPMs). For example, considering the Bayesian ridge regression at an $n$ of 75, the ERVSI for net benefit is now 0.34 and P(RVSI) $\ge$ 90% is 0.14 (Table S2), compared to 0.37 and 0.68 when the 10 noise variables were not considered (Table 4). Note that if some of the additional candidate predictors were likely to be important, then the user should then change the reference model accordingly.

#### An ‘acceptable’ sample size depends on the chosen estimands and target/assurance values

So what sample size is (minimally) acceptable? Given all the points above, the answer clearly depends on the user’s choice of estimands, their chosen model development strategy, and the assurance guarantees they want. For example, consider a user decides to use a Bayesian ridge regression for developing the pre-eclampsia model with those 10 predictor parameters. Then, a minimum sample size of 75 might be deemed acceptable for net benefit if the user targets an RVSI of at least 80%, as there is a 0.95 probability it will be between 83 and 97% (Table 4). However, if they also target an expected MAPE of 0.05, then $n$ of 75 is insufficient (mean MAPE of 0.13, Table 4) and an $n$ of 456 is preferred (mean MAPE of 0.05, Table 5). Further, if the user wants a probability of 0.9 that 0.85 $\le$ calibration slope $\le$ 1.15, to aim for well calibrated model, then an $n$ of 456 is also insufficient (probability = 0.70) and further investigation identifies an $n$ of about 1000 is needed (probability = 0.90). Even higher sample sizes may be required to tackle fairness and stability across subgroups [9, 41].

### Accounting for uncertainty in the reference model

So far, the results have focused on assuming one reference model. However, as explained in Section  A general simulation-based approach for examining required sample size, the uncertainty in the reference model can be embedded in the simulation framework. To illustrate this, consider the pre-eclampsia application again, with ten predictor parameters and allowing for four different reference models: the original (model 1) and three others (models 2 to 4). The relative predictor weights and true prevalence were fixed for all models (as stated for the model 1 in Section Applied example: simulation set-up, model development approaches and performance measures), but their true c-statistic (net benefit) were varied: 0.71 (0.38) for model 2, 0.76 (0.41) for model 1, 0.79 (0.42) for model 3, and 0.82 (0.44) for model 4. The probability of selecting each model was set at 0.1, 0.5, 0.3 and 0.1, respectively; this reflects a skewed distribution which gives lower chance of the extreme c-statistics and most weight to the original model, but also the potential for slightly larger c-statistics than before.

Focusing on model development using a frequentist logistic regression with a lasso penalty, Table 6 (for $n$ of 75 and 456) shows that the results when accounting for uncertainty in the reference model are very similar to those results when assuming the original model 1 is the truth. The key differences are a slightly larger expected c-statistic (which stems from three of the four alternative reference models having larger c-statistics), slightly wider 95% intervals for individual risks, and slightly higher assurance probabilities for the calibration slope and net benefit. However, the differences are minor. In a different example, the impact of accounting for uncertainty may be more pronounced, depending on the range of plausible reference models and the weight given to each.

**Table 6:** Summary of posterior distributions (based on 1000 samples) of the anticipated CPM performance, with and without allowing for uncertainty in the reference model (as explained in Section Accounting for uncertainty in the reference model). Performance, degradation and instability are obtained from a large evaluation dataset, for CPMs developed using frequentist logistic regression with a lasso penalty and 10 predictor parameters with either 75 or 456 participants. Degradation is examined relative to the performance of the true reference model(s)

		***Error and uncertainty of predictions from CPM***		***Calibration, discrimination and clinical utility of CPM***		
**Sample size approach**	**Sample size**	**MAPE:** mean (95% range)	**95% interval width:** mean (95% range)	**Calibration slope:** mean (95% range) P(0.9 < slope < 1.1) P(0.85 < slope < 1.15)	**C-statistic:** mean (95% range) mean degradation (95% range)	**Net benefit:** mean (95% range) ERVSI (95% RVSI range) P(RVSI > 90%)
Fully simulation-based (one reference model)	75	0.13 (0.07 to 0.18)	0.47 (0.32 to 0.71)	0.95 (0 to 3.18) 0.13 0.18	0.65 (0.50 to 0.73) −0.11 (−0.26 to −0.03)	0.37 (0.33 to 0.39) 90.4% (82.3% to 95.4%) 0.49
Fully simulation-based (multiple reference models)	75	0.12 (0.07 to 0.17)	0.49 (0.33 to 0.74)	0.94 (0 to 2.66) 0.13 0.21	0.67 (0.50 to 0.78) −0.10 (−0.29 to −0.03)	0.37 (0.35 to 0.41) 90.7% (83.9% to 96.2%) 0.54
Fully simulation-based (one reference model)	456	0.051 (0.030 to 0.074)	0.24 (0.07 to 0.43)	0.99 (0.76 to 1.33) 0.53 0.75	0.74 (0.73 to 0.75) −0.02 (−0.01 to −0.03)	0.40 (0.38 to 0.40) 97.6% (94.5% to 99.2%) 1.0
Fully simulation-based (multiple reference models)	456	0.053 (0.030 to 0.081)	0.26 (0.09 to 0.45)	0.99 (0.73 to 1.30) 0.55 0.76	0.75 (0.69 to 0.81) −0.02 (−0.03 to −0.01)	0.41 (0.38 to 0.44) 98.0% (94.9% to 99.5%) 1.0

### Computational time

The time required to undertake the fully simulation-based sample size approach will depend on various factors, including the software package being used, the model development strategy being evaluated, the number of candidate predictors, the target performance metrics of interest, and the computer processing power available. For example, using a 2021 MacBook Pro with 32GB ram, the time required to implement the fully simulation-based approach in Stata/SE to produce a row of results in Tables 4 or 5, was about 45 min for penalised logistic regression models in a frequentist framework, and about 100 min in a Bayesian framework. In general, computational time was substantially reduced when using R. For example, whereas Stata/SE took about 45 min for examining one sample size for a penalised logistic regression model in a frequentist framework, R took about 10 min.

### Results using the approach based on Fisher’s information decomposition

To help substantially reduce computational time, the approximate approaches based on Fisher’s information decomposition can also be used. For example, for the Bayesian penalised regression model, the computational time in Stata/SE was substantially reduced from 100 min (for the fully simulation-based approach) to 15 min (for the approximation based on Fisher’s information). In R it was reduced from about 30 min to 10 min.

The results (shaded rows of Tables 6 and 7, S1 and S2) suggest the approximate methods obtain similar expected values of performance and degradation to those from the fully simulation-based approach, except for the calibration slope at $n$ of 75. Expected values of calibration slope are more similar at the larger sample sizes of $n$ of 335 or 456 (the minimum recommended by *pmsampsize*); measures of uncertainty, instability and assurance also appear a closer approximation in these situations, which is as anticipated given the approximation theory is based on samples not being small (Table 8).

**Table 7:** Comparison of the fully simulation-based approach and the approximation based on Fisher’s information decomposition. Results summarise posterior distributions (based on 1000 samples) of the anticipated CPM performance, degradation and instability in a large evaluation dataset, for CPMs developed with particular modelling approaches using a sample size of 75 participants (~ 51 events) and 10 predictor parameters. Degradation is examined relative to the performance of the reference model shown in Table 3, which has a calibration slope of 1, c-statistic of 0.76, and net benefit of 0.41 in the target population. Each shaded row gives results for the approximation method (to improve computational speed) to the fully simulation-based approach of the previous row. ERVSI was very similar for the winning strategy

		***Error and uncertainty of predictions from CPM***		***Calibration, discrimination and clinical utility of CPM***		
**CPM development approach**	**Sample size approach**	**MAPE:** mean (95% range)	**95% interval width:** mean (95% range)	**Calibration slope:** mean (95% range) P(0.9 < slope < 1.1) P(0.85 < slope < 1.15)	**C-statistic:** mean (95% range) mean degradation (95% range)	**Net benefit:** mean (95% range) ERVSI (95% RVSI range) P(RVSI > 90%)
Unpenalised logistic regression (frequentist)	Fully simulation-based	0.15 (0.09 to 0.22)	0.66 (0.26 to 0.94)	0.45 (0.17 to 0.83) 0.01 0.02	0.68 (0.60 to 0.73) −0.08 (−0.16 to −0.03)	0.36 (0.30 to 0.39) 87.8% (73.9% to 96.4%) 0.42
Unpenalised logistic regression (frequentist)	Frequentist approximation via Fisher’s information decomposition	0.12 (0.07 to 0.18)	0.56 (0.29 to 0.83)	0.57 (0.34 to 0.89) 0.02 0.03	0.70 (0.60 to 0.74) −0.06 (−0.16 to −0.02)	0.36 (0.29 to 0.39) 90.3% (77.3% to 96.9%) 0.64
Ridge logistic regression (Bayesian)	Fully simulation-based	0.11 (0.06 to 0.17)	0.48 (0.28 to 0.76)	1.27 (0.35 to 4.02) 0.12 0.20	0.70 (0.62 to 0.70) −0.06 (−0.14 to −0.02)	0.37 (0.34 to 0.39) 91.9% (82.9% to 97.6%) 0.75
Ridge logistic regression (Bayesian)	Bayesian approximation via Fisher’s information decomposition	0.15 (0.08 to 0.22)	0.44 (0.31 to 0.66)	0.89 (−0.91 to 2.43) 0.15 0.23	0.61 (0.43 to 0.73) −0.15 (−0.33 to −0.03)	0.35 (0.24 to 0.39) 86.6% (60.9% to 94.2%) 0.34
Lasso logistic regression (Bayesian)	Fully simulation-based	0.13 (0.07 to 0.19)	0.59 (0.16 to 0.90)	0.53 (0.25 to 0.94) 0.03 0.04	0.69 (0.61 to 0.74) −0.07 (−0.15 to −0.02)	0.37 (0.32 to 0.39) 90.8% (80.5% to 97.5%) 0.65
Lasso logistic regression (Bayesian)	Bayesian approximation via Fisher’s information decomposition	0.12 (0.07 to 0.19)	0.55 (0.34 to 0.81)	0.65 (0.31 to 1.06) 0.06 0.11	0.68 (0.57 to 0.74) −0.08 (−0.18 to −0.02)	0.36 (0.29 to 0.39) 88.2% (68.7% to 97.5%) 0.45

**Table 8:** Comparison of the fully simulation-based approach and the approximation based on Fisher’s information decomposition. Results summarise posterior distributions (based on 1000 samples) of the anticipated CPM performance, degradation and instability in a large evaluation dataset, for CPMs developed with particular modelling approaches using a sample size of 456 participants (~ 310 events) and 10 predictor parameters. Degradation is examined relative to the performance of the reference model shown in Table 3, which has a calibration slope of 1, c-statistic of 0.76, and net benefit of 0.41 in the target population. Each shaded row gives results for the approximation method (to improve computational speed) to the fully simulation-based approach of the previous row. ERVSI was very similar for the winning strategy

		***Error and uncertainty of predictions from CPM***		***Calibration, discrimination and clinical utility of CPM***		
**CPM development approach**	**Sample size approach**	**MAPE:** mean (95% range)	**95% interval width:** mean (95% range)	**Calibration slope:** mean (95% range) P(0.9 < slope < 1.1) P(0.85 < slope < 1.15)	**C-statistic:** mean (95% range) mean degradation (95% range)	**Net benefit:** mean (95% range) ERVSI (95% RVSI range) P(RVSI > 90%)
Unpenalised logistic regression (frequentist)	Fully simulation-based	0.051 (0.030 to 0.075)	0.25 (0.05 to 0.50)	0.89 (0.69 to 1.14) 0.36 0.57	0.75 (0.73 to 0.76) −0.01 (−0.03 to 0)	0.40 (0.39 to 0.40) 97.9% (95.5% to 99.6%) 1.0
Unpenalised logistic regression (frequentist)	Frequentist approximation via Fisher’s information decomposition	0.050 (0.031 to 0.074)	0.24 (0.06 to 0.43)	0.91 (0.72 to 1.16) 0.41 0.65	0.74 (0.73 to 0.75) −0.02 (−0.03 to −0.01)	0.40 (0.39 to 0.40) 98.1% (95.2% to 99.5%) 1.0
Ridge logistic regression (Bayesian)	Fully simulation-based	0.050 (0.029 to 0.073)	0.24 (0.07 to 0.43)	1.00 (0.74 to 1.35) 0.50 0.70	0.74 (0.73 to 0.75) −0.02 (−0.03 to −0.01)	0.40 (0.39 to 0.40) 98.0% (95.9% to 99.6%) 1.0
Ridge logistic regression (Bayesian)	Bayesian approximation via Fisher’s information decomposition	0.055 (0.030 to 0.080)	0.24 (0.10 to 0.42)	1.10 (0.84 to 1.48) 0.44 0.62	0.74 (0.72 to 0.75) −0.02 (−0.04 to −0.01)	0.40 (0.39 to 0.40) 97.8% (94.4% to 99.5%) 1.0
Lasso logistic regression (Bayesian)	Fully simulation-based	0.052 (0.031 to 0.077)	0.25 (0.06 to 0.47)	0.89 (0.67 to 1.16) 0.39 0.60	0.74 (0.73 to 0.75) −0.02 (−0.03 to −0.01)	0.40 (0.38 to 0.40) 97.9% (94.8% to 99.5%) 1.0
Lasso logistic regression (Bayesian)	Bayesian approximation via Fisher’s information decomposition	0.051 (0.031 to 0.075)	0.24 (0.07 to 0.44)	0.97 (0.77 to 1.25) 0.56 0.77	0.74 (0.72 to 0.75) −0.02 (−0.03 to −0.01)	0.40 (0.39 to 0.40) 97.9% (95.4% to 99.5%) 1.0

## Discussion

This article has proposed a general approach to sample size calculations for studies developing or updating a CPM, which embeds and generalises previous proposals, allowing any predictive AI-based modelling approach for supervised learning to be considered [4–8, 10]. The work brings together various statistical concepts, methods and metrics, to provide an integrated framework that allows researchers to identify and justify (e.g., to funders) their target (minimum) sample size for participant recruitment, or to decide whether the sample size of an existing dataset is suitable. A novel contribution is directing researchers to anticipate the sampling or posterior distributions for model performance and degradation, conditional on a chosen sample size and model development strategy, in relation to a chosen set of candidate predictors and an assumed reference model. The generality of the approach stems from it allowing, in step (6), any model development (supervised learning) strategy that requires a sample of data (containing predictor and outcome values) to develop an equation, system or object (black box), which can then be used in step (8) to output (estimate) predictions for individuals in the large dataset of the target population.

As the fully-simulation based approach can be computationally intensive, we proposed using an approximation for Bayesian penalised regression models based on decomposing Fisher’s information, which serves as a useful approximation unless the effective sample size is small. In particular, the substantial reduction in computational time will help those users needing to examine many different scenarios (sample sizes, modelling strategies, number of predictors etc.), so that only a few then need to be confirmed by the fully-simulation based approach. Further research to improve computational speed (e.g., via parallelisation) and produce dedicated software packages is now needed. For now, researchers can adapt the example Stata and R code available at https://github.com/Richard-D-Riley/code and we intend to embed the approach within our *pmsampsize* package in R, Stata and Python.

Regarding the sample size to begin investigations with, we recommend using the minimum sample size recommended by the Riley et al. criteria [5], as implemented in the *pmsampsize *package [39, 40]. Our new work goes beyond the *pmsampsize* calculations, as it allows *any* model development approach (e.g., regression, ensemble methods, neural networks etc.) and *any* performance metric to be examined (e.g. calibration, discrimination, clinical utility, and overall fit), in terms of the expected value and variability of values anticipated in the target population. Further, it allows the evaluation of model degradation by making comparisons to a reference model, and by determining assurance probabilities about performance and degradation in any one realised development dataset, both overall and in subgroups (e.g., as part of fairness considerations). Assurance is more relevant to users than the expected value, as it is known that even when statistical and machine learning methods work well *on average*, they may have substantially lower performance in the single dataset being used for model development [42, 43]. In our example, we focused on performance measures considered most relevant to clinical practice, but in principle any metric could be used. Also, although our sample size calculations aim to improve the assurance of a model development study, they do not negate having to evaluate a model’s predictive performance after development. Internal and external validation approaches are essential for this purpose [44].

Deciding upon the case-mix distribution and the reference model are important aspects of our sample size approach. Indeed, the approach is most applicable when pilot or existing data are available to inform case-mix distributions, and when aiming to update or build from existing models, so that a well-justified reference model can be specified. Reference models have been used within other Bayesian prediction methods work [45], and align with Sir David Cox’s response to Breiman about why a transparent model (e.g., based on regression) is a helpful starting point for critical thinking about uncertainty [46]. We also showed how the uncertainty of the reference model can be embedded in the framework (Section Accounting for uncertainty in the reference model) [47]. Nonetheless, further research studies (e.g., simulation studies) are needed to investigate how sensitive the sample size calculations are to the choice of the reference model and any pragmatic assumptions about predictor weights in the absence of prior information (e.g., equal weighting of standardised predictors). Similarly, further research to investigate the impact of the choice of case-mix distribution is needed. Without existing (pilot) data, specifying the case-mix distribution can be a challenge, but initial results by Pavlou et al. suggest assuming conditional independence of predictors may still be a good approximation [8].

It is important to consider the specific clinical setting and to involve key stakeholders, such as patients and health professionals, when implementing our approach. This is needed to determine acceptable estimands, target values (e.g., of maximum model degradation), assurance probabilities and risk thresholds of interest. Although our applied focus was on models for healthcare, the proposed framework is broadly applicable to the development of predictive algorithms in any setting; again, the specific estimands, targets of interest and reference models will need to be tailored as such. Fairness is an especially relevant issue for CPMs and AI in healthcare. Here, we considered fairness in terms of ensuring a model’s performance and degradation is acceptable for all patient groups, for example defined by different ethnic groups or covariates reflecting protected characteristics [48]. However, we recognise that fairness is a broad topic and many other aspects of fairness exist [49].

So, in conclusion, we have proposed a general framework for examining the sample size required for CPM development and updating. We hope it encourages researchers to be pro-active in examining the sample size required to target CPMs with appropriate model performance, degradation, stability, clinical utility and fairness for their clinical setting and target population of interest.

## Supplementary Information

Supplementary Material 1.

## Data Availability

The synthetic data used in our applied example can be found at (https://github.com/Richard-D-Riley/code), alongside Stata and R code for evaluating the sample size required for the various model development strategies.
