# Supplementary material for: A general sample size framework for developing or updating a predictive algorithm: with application to clinical prediction models
Source: BMC Med Res Methodol. 2026 Apr 29;26:121. doi: 10.1186/s12874-026-02856-7 (PMC13196178; doi:10.1186/s12874-026-02856-7)
Supplement: Supplementary file 1 — Supplementary Material 1. [file 12874_2026_2856_MOESM1_ESM.pdf]

## SUPPLEMENTARY MATERIAL

*Table S1: Summary of posterior distributions (based on 1000 samples) of the anticipated CPM performance, degradation and instability in a large evaluation dataset, for CPMs developed with particular modelling approaches using a sample size of 335 participants (~241 events) and 10 predictor parameters. Degradation is examined relative to the performance of the reference model shown in* ***Error! Reference source not found.****, which has a slope of 1, c-statistic of 0.76, and net benefit of 0.41 in the target population. Each shaded row gives results for an approximation (to improve computational speed) to the fully simulation-based approach of the previous row. ERVSI was very similar for the winning strategy.*

| | | ***Error and uncertainty of predictions from CPM*** | | ***Calibration, discrimination and clinical utility of CPM*** | | |
|---|---|---|---|---|---|---|
| **CPM development approach** | **Sample size approach** | **MAPE:**<br>mean<br>(95% range) | **95% interval width:**<br>mean<br>(95% range) | **Calibration slope:**<br>mean<br>(95% range)<br>P(0.9<slope<1.1)<br>P(0.85<slope<1.15) | **C-statistic:**<br>mean<br>(95% range)<br>mean degradation<br>(95% range) | **Net benefit:**<br>mean<br>(95% range)<br>REVSI<br>(95% RVSI range)<br>P(RVSI>90%) |
| Unpenalised logistic regression (frequentist) | Fully simulation-based | 0.06<br>(0.036 to 0.087) | 0.29<br>(0.06 to 0.53) | 0.85<br>(0.63 to 1.14)<br>0.26<br>0.43 | 0.74<br>(0.72 to 0.75)<br>-0.02<br>(-0.04 to -0.01) | 0.40<br>(0.38 to 0.40)<br>97.1%<br>(94.1% to 99.3%)<br>1.0 |
| Unpenalised logistic regression (frequentist) | Frequentist approximation via Fisher's information decomposition | 0.059<br>(0.036 to 0.086) | 0.28<br>(0.08 to 0.50) | 0.88<br>(0.67 to 1.15)<br>0.31<br>0.51 | 0.74<br>(0.72 to 0.75)<br>-0.020<br>(-0.04 to -0.01) | 0.39<br>(0.38 to 0.40)<br>96.7%<br>(93.4% to 98.7%)<br>1.0 |
| Unpenalised logistic regression + heuristic shrinkage | Fully simulation-based | 0.058<br>(0.033 to 0.085) | 0.28<br>(0.09 to 0.48) | 0.98<br>(0.69 to 1.43)<br>0.40<br>0.56 | 0.74<br>(0.72 to 0.75)<br>-0.020<br>(-0.04 to -0.01) | 0.39<br>(0.38 to 0.40)<br>96.7%<br>(93.4% to 98.8%)<br>1.0 |
| Ridge logistic regression (frequentist) | Fully simulation-based | 0.056<br>(0.032 to 0.081) | 0.24<br>(0.09 to 0.42) | 1.12<br>(0.78 to 1.66)<br>0.36<br>0.52 | 0.74<br>(0.72 to 0.75)<br>-0.02<br>(-0.04 to -0.01) | 0.39<br>(0.38 to 0.40)<br>96.9%<br>(93.9% to 98.9%)<br>1.0 |
| Ridge logistic regression (Bayesian) | Fully simulation-based | 0.058<br>(0.035 to 0.087) | 0.27<br>(0.08 to 0.49) | 0.99<br>(0.69 to 1.39)<br>0.44<br>0.60 | 0.74<br>(0.72 to 0.75)<br>-0.021<br>(-0.04 to -0.01) | 0.39<br>(0.38 to 0.40)<br>96.7%<br>(93.1% to 98.7%)<br>1.0 |
| Ridge logistic regression (Bayesian) | Bayesian approximation via Fisher's information decomposition | 0.066<br>(0.036 to 0.096) | 0.26<br>(0.12 to 0.47) | 1.04<br>(0.78 to 1.47)<br>0.49<br>0.68 | 0.73<br>(0.70 to 0.75)<br>-0.028<br>(-0.06 to -0.01) | 0.39<br>(0.38 to 0.40)<br>96.0%<br>(92.4% to 98.6%)<br>1.0 |
| Lasso logistic regression (frequentist) | Fully simulation-based | 0.059<br>(0.036 to 0.088) | 0.27<br>(0.09 to 0.48) | 1.00<br>(0.69 to 1.45)<br>0.44<br>0.63 | 0.74<br>(0.71 to 0.75)<br>-0.02<br>(-0.04 to -0.01) | 0.39<br>(0.38 to 0.40)<br>96.6%<br>(93.4% to 98.6%)<br>1.0 |
| Lasso logistic regression (Bayesian) | Fully simulation-based | 0.061<br>(0.037 to 0.091) | 0.30<br>(0.07 to 0.54) | 0.85<br>(0.60 to 1.16)<br>0.30<br>0.46 | 0.74<br>(0.71 to 0.75)<br>-0.022<br>(-0.04 to -0.01) | 0.39<br>(0.38 to 0.40)<br>96.5%<br>(92.9% to 98.7%)<br>1.0 |
| Lasso logistic regression (Bayesian) | Bayesian approximation via Fisher's information decomposition | 0.059<br>(0.035 to 0.089) | 0.28<br>(0.09 to 0.50) | 0.94<br>(0.73 to 1.25)<br>0.49<br>0.67 | 0.74<br>(0.71 to 0.75)<br>-0.022<br>(-0.05 to -0.01) | 0.39<br>(0.38 to 0.40)<br>96.5%<br>(92.8% to 98.7%)<br>1.0 |
| Random forest (100 trees, depth 3) | Fully simulation-based | 0.092<br>(0.079 to 0.108) | 0.23<br>(0.16 to 0.30) | 1.53<br>(1.16 to 1.97)<br>0.01<br>0.02 | 0.72<br>(0.70 to 0.73)<br>-0.04<br>(-0.06 to -0.03) | 0.38<br>(0.36 to 0.39)<br>93.0%<br>(89.7% to 95.7%)<br>0.95 |
| Random forest (100 trees, depth 15) | Fully simulation-based | 0.115<br>(0.100 to 0.133) | 0.52<br>(0.21 to 0.71) | 0.62<br>(0.53 to 0.71)<br>0<br>0 | 0.69<br>(0.66 to 0.71)<br>-0.07<br>(-0.10 to -0.06) | 0.37<br>(0.35 to 0.38)<br>90.4%<br>(86.5% to 93.5%)<br>0.64 |

*Table S2: Summary of posterior distributions (based on 1000 samples) of the anticipated CPM performance, degradation and instability in a large evaluation dataset, for CPMs developed with particular modelling approaches using a sample size of 75 participants (~51 events) and 20 candidate predictor parameters (including 10 noise predictor parameters). Degradation is examined relative to the performance of the reference model shown in* ***Error! Reference source not found.****, which has a slope of 1, c-statistic of 0.76, and net benefit of 0.41 in the target population. Each shaded row gives results for an approximation (to improve computational speed) to the fully simulation-based approach of the previous row. ERVSI was very similar for the winning strategy.*

| | | ***Error and uncertainty of predictions from CPM*** | | ***Calibration, discrimination and clinical utility of CPM*** | | |
|---|---|---|---|---|---|---|
| **CPM development approach** | **Sample size approach** | **MAPE:**<br>mean<br>(95% range) | **95% interval width:**<br>mean<br>(95% range) | **Calibration slope:**<br>mean<br>(95% range)<br>P(0.9<slope<1.1)<br>P(0.85<slope<1.15) | **C-statistic:**<br>mean<br>(95% range)<br>mean degradation<br>(95% range) | **Net benefit:**<br>mean<br>(95% range)<br>REVSI<br>(95% RVSI range)<br>P(RVSI>90%) |
| Unpenalised logistic regression (frequentist) | Fully simulation-based | 0.24<br>(0.16 to 0.36) | 0.94<br>(0.56 to 1.0) | 0.17<br>(0 to 0.38)<br>0<br>0 | 0.63<br>(0.54 to 0.70)<br>-0.12<br>(-0.22 to -0.06) | 0.32<br>(0.25 to 0.36)<br>78.3%<br>(61.9% to 89.5%)<br>0.02 |
| Unpenalised logistic regression (frequentist) | Frequentist approximation via Fisher's information decomposition | 0.16<br>(0.12 to 0.22) | 0.73<br>(0.28 to 0.91) | 0.39<br>(0.19 to 0.56)<br>0<br>0 | 0.66<br>(0.58 to 0.72)<br>-0.10<br>(-0.18 to -0.04) | 0.34<br>(0.28 to 0.37)<br>83.4%<br>(67.6% to 92.0%)<br>0.10 |
| Unpenalised logistic regression + heuristic shrinkage | Fully simulation-based | 0.18<br>(0.11 to 0.33) | 0.84<br>(0.46 to 0.99) | 0.41<br>(-2.19 to 3.95)<br>0.04<br>0.06 | 0.62<br>(0.37 to 0.70)<br>-0.13<br>(-0.38 to -0.06) | 0.34<br>(0.28 to 0.38)<br>83.8%<br>(68.0% to 92.2%)<br>0.11 |
| Ridge logistic regression (frequentist) | Fully simulation-based | 0.13<br>(0.09 to 0.17) | 0.45<br>(0.33 to 0.64) | 2.21<br>(0 to 7.22)<br>0.14<br>0.20 | 0.67<br>(0.51 to 0.72)<br>-0.09<br>(-0.25 to -0.04) | 0.37<br>(0.33 to 0.38)<br>90.1%<br>(81.9% to 94.1%)<br>0.52 |
| Ridge logistic regression (Bayesian) | Fully simulation-based | 0.19<br>(0.11 to 0.34) | 0.86<br>(0.37 to 1.00) | 0.41<br>(0.01 to 1.21)<br>0.04<br>0.05 | 0.65<br>(0.56 to 0.70)<br>-0.11<br>(-0.20 to -0.05) | 0.34<br>(0.28 to 0.37)<br>84.0%<br>(69.5% to 92.0%)<br>0.14 |
| Ridge logistic regression (Bayesian) | Bayesian approximation via Fisher's information decomposition | 0.17<br>(0.12 to 0.22) | 0.46<br>(0.36 to 0.61) | 0.51<br>(-0.74 to 1.52)<br>0.11<br>0.15 | 0.57<br>(0.43 to 0.67)<br>-0.19<br>(-0.33 to -0.08) | 0.33<br>(0.22 to 0.37)<br>82.2%<br>(54.2% to 90.6%)<br>0.05 |
| Lasso logistic regression (frequentist) | Fully simulation-based | 0.14<br>(0.09 to 0.19) | 0.49<br>(0.36 to 0.69) | 0.93<br>(0 to 3.42)<br>0.11<br>0.16 | 0.62<br>(0.50 to 0.71)<br>-0.14<br>(-0.26 to -0.05) | 0.36<br>(0.32 to 0.38)<br>88.8%<br>(78.8% to 93.1%)<br>0.24 |
| Lasso logistic regression (Bayesian) | Fully simulation-based | 0.21<br>(0.12 to 0.34) | 0.88<br>(0.36 to 1.00) | 0.26<br>(0.02 to 0.57)<br>0<br>0 | 0.65<br>(0.57 to 0.71)<br>-0.11<br>(-0.19 to -0.05) | 0.34<br>(0.28 to 0.37)<br>82.5%<br>(69.0% to 92.1%)<br>0.093 |
| Lasso logistic regression (Bayesian) | Bayesian approximation via Fisher's information decomposition | 0.16<br>(0.11 to 0.21) | 0.69<br>(0.48 to 0.86) | 0.43<br>(0.11 to 0.72)<br>0<br>0 | 0.64<br>(0.53 to 0.71)<br>-0.12<br>(-0.23 to -0.05) | 0.33<br>(0.26 to 0.38)<br>82.1%<br>(64.0% to 92.3%)<br>0.105 |
| Random forest (100 trees, depth 3) | Fully simulation-based | 0.14<br>(0.11 to 0.17) | 0.38<br>(0.30 to 0.43) | 1.04<br>(0.54 to 1.51)<br>0.31<br>0.44 | 0.64<br>(0.57 to 0.69)<br>-0.12<br>(-0.19 to -0.07) | 0.36<br>(0.34 to 0.38)<br>89.4%<br>(83.0% to 92.3%)<br>0.37 |
| Random forest (100 trees, depth 15) | Fully simulation-based | 0.14<br>(0.11 to 0.17) | 0.47<br>(0.35 to 0.55) | 0.72<br>(0.36 to 1.01)<br>0.1<br>0.21 | 0.64<br>(0.56 to 0.68)<br>-0.13<br>(-0.20 to -0.07) | 0.36<br>(0.32 to 0.37)<br>87.7%<br>(78.4% to 92.0%)<br>0.25 |
